# Mapping short association fibre connectivity up to V3 in the human brain *in vivo*

**DOI:** 10.1093/cercor/bhae279

**Published:** 2024-07-24

**Authors:** Fakhereh Movahedian Attar, Evgeniya Kirilina, Daniel Haenelt, Robert Trampel, Kerrin J Pine, Luke J Edwards, Nikolaus Weiskopf

**Affiliations:** Department of Neurophysics, Max Planck Institute for Human Cognitive and Brain Sciences, 04103 Leipzig, Germany; Department of Neurophysics, Max Planck Institute for Human Cognitive and Brain Sciences, 04103 Leipzig, Germany; Department of Neurophysics, Max Planck Institute for Human Cognitive and Brain Sciences, 04103 Leipzig, Germany; Department of Neurophysics, Max Planck Institute for Human Cognitive and Brain Sciences, 04103 Leipzig, Germany; Department of Neurophysics, Max Planck Institute for Human Cognitive and Brain Sciences, 04103 Leipzig, Germany; Department of Neurophysics, Max Planck Institute for Human Cognitive and Brain Sciences, 04103 Leipzig, Germany; Department of Neurophysics, Max Planck Institute for Human Cognitive and Brain Sciences, 04103 Leipzig, Germany; Felix Bloch Institute for Solid State Physics, Faculty of Physics and Earth System Sciences, Leipzig University, 04103 Leipzig, Germany; Wellcome Centre for Human Neuroimaging, Institute of Neurology, University College London, London WC1N 3AR, United Kingdom

**Keywords:** retinotopy, superficial white matter, tractography, U-fibres, validation

## Abstract

Short association fibres (SAF) are the most abundant fibre pathways in the human white matter. Until recently, SAF could not be mapped comprehensively *in vivo* because diffusion weighted magnetic resonance imaging with sufficiently high spatial resolution needed to map these thin and short pathways was not possible. Recent developments in acquisition hardware and sequences allowed us to create a dedicated *in vivo* method for mapping the SAF based on sub-millimetre spatial resolution diffusion weighted tractography, which we validated in the human primary (V1) and secondary (V2) visual cortex against the expected SAF retinotopic order. Here, we extended our original study to assess the feasibility of the method to map SAF in higher cortical areas by including SAF up to V3. Our results reproduced the expected retinotopic order of SAF in the V2–V3 and V1–V3 stream, demonstrating greater robustness to the shorter V1–V2 and V2–V3 than the longer V1–V3 connections. The demonstrated ability of the method to map higher-order SAF connectivity patterns *in vivo* is an important step towards its application across the brain.

## Introduction

Short association fibres (SAF) connect mainly adjacent cortical areas in the superficial white matter of gyrencephalic brains (Schüz et al. 2002). SAF are thin and short with estimated mean layer thickness 1.5 mm and length 3–30 mm in the human brain (Schüz et al. 2002). Dedicated non-invasive methods using high-spatial-resolution magnetic resonance imaging (MRI) are required to comprehensively map the SAF *in vivo* (Song et al. 2014). Because our ground-truth knowledge of SAF in the human brain is currently limited, validating maps of SAF obtained *in vivo* is challenging and must be carefully addressed.

Patterns of SAF connectivity in the primate early visual processing stream are implied by the retinotopic organisation principle (Inouye 1909;^18,19^). Retinotopic organisation suggests the presence of strong SAF connections between points in the primary (V1), secondary (V2), and tertiary (V3) visual cortex if and only if they represent the same retinotopic coordinates in the visual field. Short-range feedforward and feedback white matter connections have been demonstrated between V1–V2, V2–V3, and V1–V3 using tracer injections in non-human primates (Felleman and Van Essen 1991). Using this *a priori* knowledge of SAF connectivity patterns, the *in vivo* methods designed to map the SAF pathways can be validated.

In our previous work, we presented a multi-modality MRI framework designed to map the structural SAF connectivity in humans *in vivo* and validated it by demonstrating the expected retinotopic order of V1–V2 SAF connectivity (Movahedian Attar et al. 2020). To validate our approach beyond the connections of two cortical areas, SAF connectivity patterns may be mapped from V1 over V2 up to V3.

We used sub-millimetre (0.8 mm) isotropic spatial resolution diffusion weighted imaging (DWI) tractography to map the SAF connectivity patterns between V1, V2, and V3, which we mapped using functional MRI (fMRI) retinotopic mapping (Movahedian Attar et al. 2020). Our group connectivity matrices showed retinotopic V2–V3 and V1–V3 SAF *in vivo*, where patterns for the nearest-neighbour connectivity (V1–V2 and V2–V3) were stronger and more robust than the next-neighbour V1–V3 connectivity.

## Materials and methods

The data were acquired as part of a previous study (Movahedian Attar et al. 2020) and the processing pipeline and connectivity analysis were largely similar. For more detailed information about data acquisition, preprocessing, and analysis, we refer the reader to the original publication (Movahedian Attar et al. 2020). The minor modifications in the data processing pipeline are described below.

### Participants and DWI acquisition

Briefly, we acquired ultrahigh-spatial-resolution DWI *in vivo* with a slab covering the occipital cortex on a 3T Connectom MRI scanner (Siemens Healthcare, Erlangen) (Setsompop et al. 2013; Jones et al. 2018) and using a 32-channel radio-frequency head coil (voxel size 0.8 mm isotropic, b-values 800 and 1800 s/mm^2^, slab containing 62 oblique near-axial slices placed to cover the V1 and V2 cortex, 60 diffusion-encoding directions per diffusion-weighted shell, 25 interleaved non-diffusion-weighted volumes acquired after every 10 DWI volumes, anterior–posterior phase encoding, echo time $\text{(TE)}=66$ ms, repetition time $\text{(TR)}=8900$ ms, diffusion gradient length $(\delta )=6$ ms, spacing between diffusion gradient pulses $(\Delta )=22$ ms, maximum gradient amplitude 188 mT/m). To correct susceptibility-related distortions, an additional b = 0 s/mm^2^ volume acquired immediately before the DWI series with reversed phase encoding was used.

Because the study in Movahedian Attar et al. (2020) was designed to investigate V1 and V2, the acquired DWI slab contained coverage of V3 only in a subset of cases. The present study included data from 18 hemispheres (9 right, 9 left) from 11 participants in the original cohort for which V1, V2, and V3 were contained in the DWI slab and could all be mapped using fMRI retinotopic mapping.

Functional and structural MRI were acquired on a 7 T scanner (Siemens Healthcare, Erlangen) to robustly map V1, V2, and V3 (Sereno et al. 1995; Engel et al. 1997) and allow us to reconstruct the cortical surfaces with FreeSurfer software package (Fischl 2012), respectively. Visual stimulation in fMRI retinotopic mapping consisted of a phase-encoded paradigm (Sereno et al. 1995; Engel et al. 1997) with a contrast-reversing checkerboard stimulus, which was restricted to expanding/contracting rings along the visual field eccentricity and a clockwise/anticlockwise rotating ray along polar angle directions (Movahedian Attar et al. 2020).

### Functional MRI

#### Smoothing eccentricity and polar angle maps

**Fig. 1:**
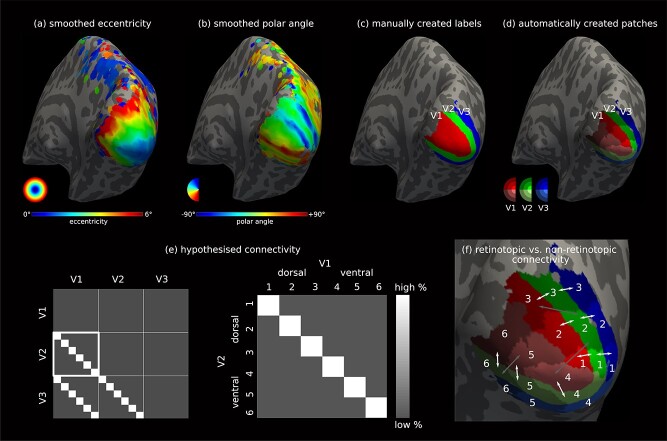
SAF connectivity mapping based on fMRI retinotopic mapping of V1, V2, and V3 enables partial validation *in vivo*. For a single representative hemisphere, smoothed (a) eccentricity and (b) polar angle maps of visual field coordinates obtained with fMRI overlaid on the inflated cortical surface. (c) Manually delineated V1, V2, and V3 labels based on the retinotopy maps. (d) The six automatically segmented sub-areas within each of V1, V2, and V3 based on the retinotopy maps, equally spaced in the visual field. (e) The corresponding hypothesised connectivity matrix based on the retinotopic organisation principle. Example $6\times 6$ matrix for V1–V2 connectivity. (f) Definition of six retinotopic sub-areas for each of V1, V2, and V3 together with retinotopic (white arrows) and non-retinotopic (grey arrows) connectivity.

To mitigate the effects of acquisition noise when segmenting V1, V2, and V3 into their respective sub-areas based on the retinotopic maps, we updated our original pipeline to smooth the eccentricity and polar angle phase maps on the reconstructed cortical surface. For each vertex on the surface, the mean phase value was computed based on a local neighbourhood (Hagler et al. 2006) defined around it. Only the vertices with reliable functional signal, determined using an arbitrary signal-to-noise ratio (SNR) threshold of 5, were included. SNR was computed as the magnitude of the signal at the stimulus frequency, divided by the standard deviation of the frequency spectrum of the fMRI time-series. The process was repeated 4 times to achieve sufficient smoothing of eccentricity (Fig. 1a) and polar angle (Fig. 1b) maps.

#### Delineating V1, V2, and V3 borders

The functionally defined areal borders of V1, V2, and V3 represented the meridians of the visual field and were identified by fMRI retinotopic mapping. The V1–V2 border corresponded to the representation of the vertical meridian and the V2–V3 border corresponded to the representation of the horizontal meridian. V1, V2, and V3 functional borders were delineated manually along the respective meridians using the smoothed retinotopic maps on the inflated cortical surface (Fig. 1c).

### Diffusion MRI

#### Mapping fibre pathways using probabilistic tractography

DWI were distortion corrected for noise, Gibbs ringing, bulk head motion, and geometric distortions as in our previous work (Movahedian Attar et al. 2020) using FSL (v5.0.9) (Smith et al. 2004; Woolrich et al. 2009; Mark et al. 2012), ANTS (v2.2.0) (Avants et al. 2009), gradunwarp toolbox (v1.1.0) [https://github.com/Washington-University/gradunwarp] (Sotiropoulos et al. 2013), and MRtrix3 (v3.0.0) (Tournier et al. 2019). DWI analysis was performed using MRtrix3 (v3.0.0). First, we created fibre orientation maps (ODF) using a multi-tissue decomposition approach (Dhollander et al. 2016). Second, we modified our original DWI probabilistic tractography approach (Movahedian Attar et al. 2020) to use a cortical instead of whole-brain seeding strategy for tractography. Using cortical seeding significantly reduced the number of generated streamlines, which significantly reduced the tractogram file size and thus, allowed us to use a higher seed resolution of ${4 \times 4 \times 4}$ per voxel for more robust tracking between adjacent cortical areas.

To generate the seed mask, we used the V1, V2, and V3 labels created based on fMRI retinotopic mapping and transformed to DWI space. Other tracking parameters from our original study were: curvature threshold of 30$^{\circ }$, seed and tracking ODF amplitude threshold of 0.1, tractography step size of 0.2 mm and reconstructed streamline lengths of 3–120 mm.

#### Preparing the tractography streamlines

In our previous work, we transformed the V1 and V2 functional labels from surface to DWI volume space and mapped the SAF connectivity by assigning V1–V2 streamlines as those traversing both V1 and V2. Here, to enable more precise connectivity mapping, we projected the streamlines to the cortical surface representing the grey/white interface (where no overlaps between the manually delineated V1, V2, and V3 were allowed) using a non-linear deformation field computed for each hemisphere using the ANTs software package (Avants et al. 2009). The nonlinear deformation field aligned the diffusion and structural MRI and accounted for the differences in their geometric distortion patterns.

A streamline connected two cortical areas if it terminated within both on the cortical surface. We defined the terminations as the first and last coordinates of the streamline within the cortex and obtained their precise locations using an in-house developed python script [https://github.com/dchaimow/streamline_positions_from_surfaces]. The script identified the surface vertices surrounding the first and last streamline coordinates in the triangular surface mesh. A streamline was taken to connect two cortical regions if its associated vertices on both ends were a subset of the vertices of the respective cortical areas. We removed the streamlines with more than 80 % length running inside the cortex to avoid bias from intra-cortical fibre pathways.

### SAF connectivity analysis

#### Creating the connectivity matrices

Six cortical sub-areas were defined within each of V1, V2, and V3 (Fig. 1d). We updated our original pipeline to create these sub-areas automatically in surface space using the V1, V2, and V3 labels and the smoothed polar angle and eccentricity maps as follows. First, each cortical label was divided into two segments, corresponding to the upper and lower visual hemifields in the ventral and dorsal hemispheres, respectively. Second, each polar angle segment was divided into three additional segments in the eccentricity direction. Eccentricity was normalised to the [0,1] interval and subsequently divided at 33 % intervals, resulting in three eccentricity segments that were equally spaced in the visual field domain. These corresponded approximately to the cortical representations of visual field eccentricity in the ranges 0–2$^{\circ }$, 2–4$^{\circ }$, and 4–6$^{\circ }$. Because the minimum value of eccentricity mapped in our fMRI experiment was 0.89$^{\circ }$, the first segment was smaller in size across hemispheres (see Supplementary Fig. S4).

Connectivity was mapped between each pair of cortical segments, or sub-areas, in V1, V2, and V3, resulting in an $18\times 18$ connectivity matrix for each hemisphere (Fig. 1e). The connectivity index in each element of the matrix reflected the relative connectivity strength between the corresponding pair of sub-areas in V1, V2, and V3. We computed the connectivity indices for each unique element of the symmetric connectivity matrix (omitting the diagonal elements, which represented connectivity detected within each cortical sub-area, that is, between a sub-area and itself (self connections)). The relative connectivity strength was computed for each pair of sub-areas as the total number of streamlines detected between them divided by the total number of all streamlines detected between all pairs (excluding self connections). We reported the resulting connectivity as % connectivity matrices.

The corresponding closeness matrix was created for each connectivity matrix in order to assess the effect of tractography distance bias (Ercsey-Ravasz et al. 2013; Donahue et al. 2016) on the mapped connectivity strengths. Here, reciprocals of the streamline lengths were used as a proxy measure of closeness between pairs of cortical sub-areas. For each pair of sub-areas, the mean over the reciprocals of all streamlines connecting them was given in each element of the closeness matrix.

#### Analysing the group-average retinotopic order

SAF retinotopic organisation was investigated at the group level. For each element in the $18\times 18$ connectivity matrix, we computed the mean connectivity strength across all hemispheres resulting in the group-averaged % connectivity matrix. The matrix consisted of inter-area V1–V2, V2–V3, and V1–V3 and intra-area V1–V1, V2–V2, and V3–V3 connectivity. In the intra-area case the diagonals correspond to self connections and were set to zero as mentioned above. We indicated the sub-areas by the numbers 1–6 where 1–3 were dorsal and 4–6 were ventral for each of V1, V2, and V3, as shown in Fig. 1(f). Examples of intra-area connectivity are connections between sub-areas 1 and 4 in V1, or sub-areas 5 and 6 in V2; and examples of inter-area connectivity are connections between sub-area 1 in V1 and sub-area 1 in V2, or sub-area 5 in V1 and sub-area 6 in V2.

To investigate the retinotopic organisation of the SAF we used the inter-area connectivity only. According to retinotopic organisation, strong connectivity was expected between the retinotopically corresponding sub-areas of V1, V2, and V3 indicated by the same number between 1 and 6, e.g. between sub-area 1 in V1 and sub-area 1 in V2 or sub-area 5 in V2 and sub-area 5 in V3, as indicated in Fig. 1(f) by the white arrows. These appeared in the diagonal elements of the $6\times 6$ connectivity matrices for V1–V2, V2–V3, and V1–V3 SAF as shown in Fig. 1(e). On the other hand, no connectivity was expected between the retinotopically non-corresponding sub-areas of V1, V2, and V3, e.g. between sub-area 5 in V2 and sub-area 3 in V3 or sub-area 2 in V1 and sub-area 6 in V3, as indicated in Fig. 1(f) by the grey arrows. These appeared in the off-diagonal elements of the $6\times 6$ connectivity matrices. We introduced the ratio of total retinotopic to non-retinotopic % connectivity as an index for retinotopic organisation, which we computed separately for each $6\times 6$ matrix. The corresponding group-average closeness matrix was also created. Given the absence of supporting evidence in the literature on the existence of intra-area SAF connections, we considered all the detected V1–V1, V2–V2, and V3–V3 connectivity false-positives. We nevertheless reported the contributions of these connections to the $18\times 18$ connectivity matrix to better understand the performance of our *in vivo* method.

### Statistical analysis

We assessed the robustness of our method using statistical analysis on the retinotopic order. Assuming all of the hemispheres were statistically independent, we performed a separate statistical test for each element of the group connectivity matrix over the connectivity matrices of all the hemispheres. Our null hypothesis was that connectivity was driven purely by chance, that is, by spurious tractography artefacts including the length bias.

Streamline tractography is biased towards detecting shorter streamlines (Ercsey-Ravasz et al. 2013; Donahue et al. 2016; Schilling et al. 2018), resulting in a length bias. The adjacency requirement of retinotopy (see Fig. 1f) suggests generally shorter retinotopic than longer non-retinotopic connections. This is especially true for the nearest-neighbour connectivity (V1–V2 and V2–V3) for which the retinotopically corresponding cortical sub-areas are also anatomically adjacent, resulting in shorter connection distances between two regions on the cortical surface. Therefore, the *in vivo* framework is inherently biased towards detecting the expected and against detecting some of the unexpected connectivity.

To establish a null hypothesis that models the length bias effects, we performed a probabilistic streamline tractography on an isotropic orientation distribution map (Morris et al. 2008). Using this approach, streamlines are generated by taking random walks within this isotropic field, instead of sampling the underlying fibre ODF. This will capture the contributions of length bias to the mapped connectivity patterns in the absence of fibre orientation information. We used the ‘NullDist2’ algorithm in MRtrix3 (v3.0.0) to generate these ”null streamlines”. For fair comparison against our fibre ODF mapped connectivity, we restricted null distribution tractography to the white matter mask defined by a mask of fibre ODF amplitudes greater than 0.1, consistent with our fibre ODF tracking. We also used the same curvature threshold, length threshold, and seed resolution. Null distribution tractography was repeated 10 times per hemisphere to obtain precise estimates of the null distribution of streamline terminations in the cortex.

Matrices of absolute streamline counts were generated for connectivity mapped using fibre ODF tractography and each repetition of null distribution tractography. In the following, we use the per-hemisphere mean matrices from null distribution tractography. Two statistical tests were performed for each matrix element at the 95 % significance level ($P<0.05$).

First, following the approach described in Morris et al. (2008), the null hypothesis was that the observed count $N_{ij}^{h}$ from matrix element $ij$ and hemisphere $h$ was drawn from a per-hemisphere Poisson distribution with mean given by the mean of the respective null distribution tractography count, $\mu _{ij}^{h}$, i.e. 


(1) \begin{align*}& N_{ij}^{h} \sim \text{Pois}(\mu_{ij}^{h}).\end{align*}

The known result that the sum of independent Poisson variables is distributed as a Poisson variable with mean equal to the sum of the means of the variables, that is, 


(2) \begin{align*}& \sum_{h} N_{ij}^{h} \sim \text{Pois}\left(\sum_{h} \mu_{ij}^{h}\right),\end{align*}

was used to compute a one-tailed $P$-value for the observed sum for each matrix element.

Second, to check the robustness of our findings, we performed a paired one-tailed $t$-test for each element of the ODF count matrix relative to the per-hemisphere mean of the null distribution counts over the hemispheres. This analysis does not make the assumption that the counts are necessarily Poisson distributed, instead making the weaker assumption that the mean of the paired differences is Gaussian distributed. Further, while the Poisson test inherently assumes that each $\mu _{ij}^{h}$ is an unbiased estimate of the per-hemisphere mean, the pairing in this case will mitigate random effects of inter-individual differences in tractography performance shared between the null and fibre ODF counts.

Based on the detected significant connectivity, we reported the sensitivity and specificity of the method to map retinotopic connectivity for the $18\times 18$ and each of the $6\times 6$ group connectivity matrices, separately. Sensitivity was defined as the ratio of detected (true positive) retinotopic to all expected retinotopic connectivity. Specificity was defined as the ratio of all zero (true negative) non-retinotopic connectivity to the total number of all possible non-retinotopic connectivity:


(3) \begin{align*} & \text{sensitivity} = \frac{\text{non-zero retinotopic matrix elements}}{\text{all retinotopic matrix elements}} \end{align*}

(4) \begin{align*} & \text{specificity} = \frac{\text{zero non-retinotopic matrix elements}}{\text{all non-retinotopic matrix elements}} \end{align*}

## Results

### SAF geometries vary up to V3 *in vivo*

**Fig. 2:**
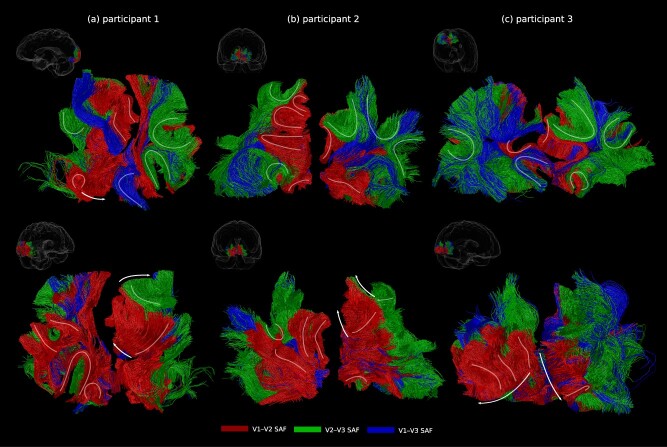
SAF were mapped up to V3, showing primarily U-shapes and V-shapes *in vivo*. SAF trajectories are shown for three representative participants (6 out of the 18 hemispheres included in the study) on two different views (top and bottom rows) on which SAF geometries could be visualised best. SAF geometries are highlighted by the manually drawn curved pathways, and arrows indicate their orderly spatial arrangement forming arrays.

SAF geometries were mapped up to V3. Figure 2 shows SAF trajectories mapped for six representative hemispheres from three participants, showing both V-shaped and the better known U-shaped SAF. In some regions, the SAF appeared as arrays of parallel running pathways (arrows in Fig. 2). Our observation in the occipital lobe pointed to potentially diverse SAF shapes across the human brain.

### SAF retinotopic order was shown up to V3 *in vivo*

**Fig. 3:**
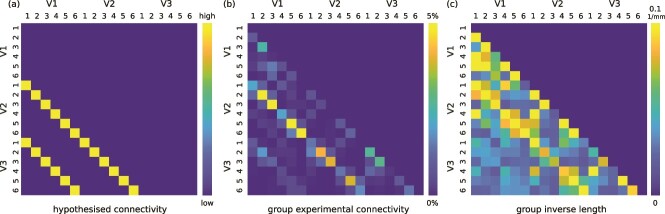
SAF retinotopic order was shown up to V3 *in vivo*. (a) Connectivity patterns hypothesised based on the retinotopic organisation principle. The highlighted elements indicate the retinotopic connections, which are “nearest-neighbours” for V1–V2 and V2–V3 and “next-neighbours” for V1–V3 connectivity, respectively. (b) The group-averaged experimental SAF connectivity matrix showing inter-area V1–V2, V2–V3, and V1–V3 SAF as well as intra-area connectivity between the different sub-areas within V1, V2, and V3. (c) The corresponding group-averaged closeness matrix computed based on the reciprocals of the streamline lengths.

Our group-averaged % connectivity matrix demonstrated SAF retinotopic order up to V3 (Fig. 3b), assessed against the corresponding hypothesised connectivity patterns (Fig. 3a). However, non-retinotopic connectivity as well as unexpected intra-area connectivity were also detected. The strongest retinotopic order was observed for V1–V2 connectivity with retinotopic and non-retinotopic contributions of 78.2 and 21.8 % (ratio = 3.6), respectively, followed by V2–V3 with 69.0 and 31.0 % (ratio = 2.2). The weakest retinotopic order was observed for V1–V3 connectivity with 61.8 and 38.2 % (ratio = 1.6). The overall retinotopic order computed based on all the inter-area connectivity was given by retinotopic and non-retinotopic contributions of 73.6 and 26.4 % (ratio = 2.8), respectively.

Based on the corresponding group closeness matrix, we observed on average shorter retinotopic and longer non-retinotopic connections (Fig. 3c). For V1–V3, the retinotopic connections appeared almost as long as the non-retinotopic ones, potentially because of the longer distance between the next-neighbour V1 and V3 cortical areas. We quantified the mean lengths (mean$\pm $standard deviation) of SAF trajectories as follows. The retinotopic estimates were $12.7\pm 5.6$ mm for V1–V2, $17.2\pm 8.3$ mm for V2–V3, and $25.0\pm 7.3$ mm for V1–V3, respectively. The non-retinotopic estimates were $17.0\pm 9.5$ mm for V1–V2, $22.9\pm 11.5$ mm for V2–V3, and $29.4\pm 9.5$ mm for V1–V3, respectively. The on average longest V1–V3 connections (next-neighbour) contributed only 8 % of the total detected connectivity in the $18\times 18$ group connectivity matrix. The on average shorter V1–V2 and V2–V3 connectivity (nearest-neighbour) contributed 45 and 24 % of the total detected connectivity, respectively. The remaining 23 % corresponded to the intra-area connectivity detected between the sub-areas within V1, V2, and V3 (8.9 % V1–V1, 3.6 % V2–V2, and 10.5 % V3–V3). Some of these unexpected connections were as short as the retinotopic SAF. Our observations pointed to the presence of a length bias in the mapped connectivity patterns.

### Sensitivity and specificity of *in vivo* SAF connectivity mapping

We assessed the robustness of our *in vivo* method to reproduce SAF retinotopic order in the presence of experimental biases and errors as follows. We characterised the sensitivity and specificity of the method for detecting retinotopic SAF based on the statistically significant SAF connectivity (95 % significance). Statistical significance was assessed against a null connectivity model capturing the length biases of tractgraphy using a Poisson test and paired $t$-test. It allowed us to identify the biological SAF connectivity, that is, connectivity that could not be explained solely by the modelled tractography biases and errors.

**Fig. 4:**
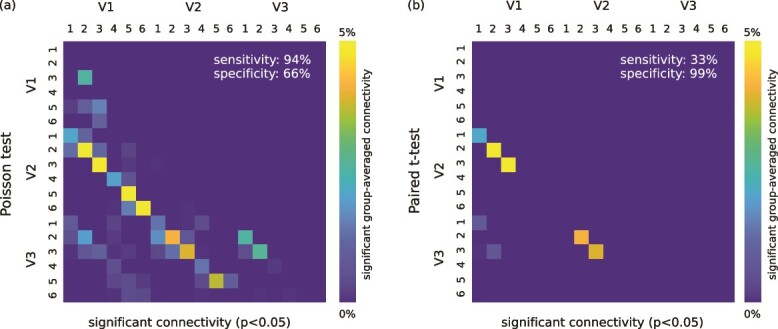
Group level analysis of SAF connectivity up to V3 to assess the sensitivity and specificity of the *in vivo* method to map the SAF. (a) Test assuming hemisphere-specific Poisson null distributions of counts between retinotopically corresponding sub-areas 1–6 (see Fig. 1) in V1, V2, and V3 cortical areas. Group averaged % connectivity is shown for each pair of sub-areas for which streamline counts significantly exceeded those in null tractography at 95 % significance level. (b) Paired $t$-test on the ODF and null streamline counts at 95 % significance level. Significant connections are shown as per (a). The estimated sensitivity and specificity are shown.

Our *in vivo* method was overall highly sensitive (94 %) but only moderately specific (66 %) to retinotopic connections ($P<0.05$) under the Poisson test (Fig. 4a). The highest possible sensitivity (100 %) was observed for V1–V2 and V1–V3 SAF and specificity was comparable between the different cases (70 %, 67 %, 63 % for V1–V2, V2–V3, and V1–V3 SAF, respectively).

The paired $t$-test (Fig. 4b), which mitigates the random effects of inter-individual differences in tractography performance, showed significantly reduced sensitivity (33 % for $P<0.05$ and 17 % for $P<0.01$) and reached its lowest value for the longest next-neighbour V1–V3 connectivity (17 % for $P<0.05$ and 0 % for $P<0.01$) and V2–V3 connectivity (0 % for $P<0.01$). Specificity to retinotopic SAF was enhanced significantly (99 %). The results of our $t$-test point to its lower sensitivity, likely explained also by hemisphere-specific differences in ODF tractography, e.g. from local differences in gyral bias and unresolved crossing fibre patterns (see Discussion).

## Discussion

Development of non-invasive methods that accurately and robustly map the SAF across the living human brain is essential. Here, we extended the validation of our *in vivo* method to map SAF connectivity patterns (Movahedian Attar et al. 2020) beyond the connections of two cortical regions. SAF connectivity patterns, previously mapped only between V1 and V2 (Movahedian Attar et al. 2020), were mapped up to V3 in the human early visual processing stream. Our results obtained by combining sub-millimetre resolution DWI tractography with fMRI retinotopic mapping of V1, V2, and V3 demonstrated the expected retinotopic pattern of SAF connectivity also in the V2–V3 and V1–V3 processing streams. By providing further validation of the method beyond a single pair of cortical regions, we demonstrated the method’s ability to map SAF connectivity across the brain *in vivo*. Our work is an important step towards creating a comprehensive map of these shortest components of the human white matter connectome across the brain.

SAF in the occipital lobe have historically been mapped in the human brain in the *post mortem* works of Sachs and Sachs (1892) and Dejerine and Dejerine-Klumpke (1895). A number of these fibres were recently reproduced using DWI tractography *in vivo* (Bugain et al. 2021). In the early visual cortex, SAF connecting V1–V3 have been shown using tracer injections in non-human primate brains (Felleman and Van Essen 1991). Comparison of our SAF mapping to results from a human brain atlas of reproducible short fibre bundles created based on DWI tractography of Human Connectome Project data *in vivo* (Labra-Avila et al. 2020) showed qualitative agreement in the V1, V2, and V3 cortex (see details of comparison in Supplementary section S.2). SAF connecting V1 up to V3 were mapped in the atlas and corresponded roughly to the anatomical locations of SAF in our study. Unlike in our study, where the streamlines were grouped based on the functional specialisation of cortical regions they connect, bundles in the atlas were created based on the common anatomical locations of their streamlines. This approach is fundamentally different from the one presented in our study, thus, limiting the possibility for precise and quantitative comparison.

We mapped the organisation of SAF pathways connecting V1, V2, and V3 for the first time *in vivo*. Our results showed diverse shapes of SAF trajectories consistent with Movahedian Attar et al. (2020), showing both U- and V-shaped trajectories distinguished by their higher and lower gyrification (or bending) angles, also consistent with Chauvel et al. (2022). Further, the SAF exhibited orderly spatial arrangements (see arrows in Fig. 2 and a quantitative description in Movahedian Attar et al. (2024)), supporting the overlying cortical retinotopic organisation. Beyond early visual processing, U-fibres with orderly organisation have also been suggested in the primary sensory-motor cortex along the central sulcus using DWI tractography (Pron et al. 2021) and U-fibres with pyramid-shaped crossings have been shown in temporal (Shinohara et al. 2020) and frontal (Catani et al. 2011) lobes using DWI tractography and *post mortem* dissections. Together with our findings in the occipital lobe, these observations point to the potentially diverse shapes and organisations of SAF pathways resulting in their potentially specific roles in information transfer across the brain. Therefore, as also pointed out in Movahedian Attar et al. (2020), the application of geometric filters to map the SAF across the brain should be revisited as these may result in significant false-negative occurrences.

SAF in early visual processing stream are expected to follow retinotopic organisation (Inouye 1909;^18,19^). Our results demonstrated the expected retinotopic pattern of SAF connectivity up to V3. The strongest retinotopic order was observed for the nearest-neighbour V1–V2 (78.2 % retinotopic and 21.8 % non-retinotopic connectivity, values consistent with findings reported in Movahedian Attar et al. (2020)) followed by V2–V3 (69.0 and 31.0 %), and then the next-neighbour V1–V3 (61.8 and 31.0 %) connectivity.

Our analysis demonstrated on average lower % connectivity for retinotopic connections detected between sub-areas 1 and 4, which corresponded to the cortical representations of the central visual field close to the occipital pole. This observation could be explained by the relatively smaller sizes of these sub-areas as mapped by our fMRI retinotopic analysis setup (see Supplementary Fig. S4). Alternatively, it could be explained by less reliable tractography in the tight and tortuous white matter junctions due to the specific cortical geometry of the occipital pole. Both could result in fewer detected streamlines.

Although the mapped connectivity strength and length were correlated (Fig. 3, Supplementary Fig. S1), the observed retinotopic patterns could not be explained solely by this length bias of tractography (Ercsey-Ravasz et al. 2013; Donahue et al. 2016). We mapped the contributions of length bias to the detected connectivity using a null distribution connectivity mapping approach (Morris et al. 2008). Compared to previous applications of models of tractography length bias (Ercsey-Ravasz et al. 2013; Donahue et al. 2016; Delettre et al. 2019), which assume an inverse exponential relationship between connectivity strength and length, our approach was model-free and based solely on the tractography algorithm (Morris et al. 2008), giving an empirical, thus, more realistic characterisation of the length bias per hemisphere.

A joint tracer injection and DWI tractography study performed in macaque brain showed the absence of false positive connections (Maier-Hein et al. 2017; Jeurissen et al. 2019) for path lengths $\le $ 20 mm (Donahue et al. 2016). The incidence of false positive streamlines increased with the path length (Donahue et al. 2016). Based on this finding, retinotopic order mapped in the V1–V2 stream was most precise because of the relatively short mean lengths of its streamlines ($12.7\pm 5.6$ mm retinotopic and $17.0\pm 9.5$ mm non-retinotopic), below the reported threshold of 20 mm. The precision could be lower for the longer V2–V3 and V1–V3 connectivity ($17.2\pm 8.3$ mm and $25.0\pm 7.3$ mm retinotopic, and $22.9\pm 11.5$ mm and $29.4\pm 9.5$ mm non-retinotopic, respectively). This finding cannot be validated further here because of a lack of corresponding quantitative knowledge of connectivity strengths and their respective pathway lengths in the human brain.

The analysis based on the assumption of Poissonian statistics demonstrated robustness of the method to map the retinotopic patterns of SAF connectivity. The alternative $t$-test approach was less sensitive, potentially highlighting the general limitations of ODF tractography, e.g. gyral bias and unresolved fibre crossings. Also, most of the detected (but unexpected) intra-area connectivity were found to be insignificant at the group level. Our overall observations supported the biological plausibility of the mapped retinotopic order of SAF connectivity up to V3 *in vivo* at the group level.

Our method was highly sensitive to retinotopic connectivity (94 % for all connectivity) with moderate specificity (70 %, 67 %, 63 % for V1–V2, V2–V3, and V1–V3, respectively) estimated for significant SAF connectivity ($P<0.05$). It could not be established whether the overall lower contribution of V1–V3 SAF (8 %) to the total detected connectivity was a by-product of the tractography length bias or because V1–V3 SAF were less abundant compared to V1–V2 and V2–V3 SAF. The lower retinotopic order of V1–V3 SAF could also potentially point to other sources of bias in the method or the underlying biology. We could not validate this interpretation further because quantitative knowledge of SAF connectivity up to V3 in the human brain is currently not available. Development of SAF connectivity models using *ex vivo* fibre quantification methods, for instance based on advanced histological techniques such as CLARITY (Morawski et al. 2017), may help us to establish the necessary ground-truth for the relative SAF connectivity strengths and path lengths in the early visual processing stream.

### Limitations

Our analysis was restricted by a number of biases and errors, which were previously described in Movahedian Attar et al. (2020). Length and gyral bias of tractography remain important obstacles in the way of obtaining accurate and comprehensive quantitative maps of SAF pathways *in vivo*. We discussed the length bias above. Gyral bias describes the tendency of tractography to propagate the streamlines in the direction of gyral crowns rather than the sulcal walls and fundi (Van Essen et al. 2014; Schilling et al. 2018). Recent work has shown that advanced surface-based tractography could not alleviate the gyral bias challenge *in vivo* (Shastin et al. 2022). Fibre modelling based on constrained spherical deconvolution with multiple response functions (De Luca et al. 2022) has the potential to improve tractography, but has not yet been tailored for application in the SAF. Similarly, microstructure-informed tractography (^5,6^; Girard et al. 2017) based on complementary quantitative MRI techniques, which may help to unravel quantitative aspects of connectivity *in vivo*, is challenging in the superficial white matter because of a lack of robust MRI co-alignment methods at sub-millimetre spatial resolutions as well as a lack of modelling approaches that can disentangle the complex fibre arrangements in this region (Reveley et al. 2015).

Gyral bias may have resulted in the misclassification of connections by shifting their precise terminations along the cortical boundary. Affected locations are where the areal borders connecting V1, V2, and V3 also fall near the crowns of gyri. Although the relatively large size of the individual elements of the connectivity matrices will have largely mitigated the effects of gyral bias in our analysis, detailed analysis on a fine-grained scale may be affected. Such fine-grained analysis is necessary because the limited and arbitrarily defined size of the cortical sub-areas could exacerbate the misclassification of connectivity. Future research could address this limitation to obtain more precise maps of the SAF retinotopic order *invivo* (see for example Movahedian Attar et al. 2024).

The potential impact of gyral bias on our $t$-test hypothesis testing is worth mentioning. Tractography based on a null distribution map samples both cortical gyri and sulci with equal probability unlike the fibre distribution map, which is affected by a gyral bias. Given the relatively large sizes of our cortical patches, streamline counts are averaged across both sulci and gyri in favour of the null case. The combination of higher resolution cortical seeding, which is expected to differentially benefit ODF tractography (Morris et al. 2008) and smaller patch sizes, which could separate sulcal/gyral regions might improve our observations in favour of ODF tractography. This possibility could not be explored further because of the extremely long computation times required to process the larger tractograms created using higher seed resolutions.

Application of null tractography in the superficial white matter may be affected by cortical folding patterns and the tight white matter junctions they make between adjacent cortical segments. Using cortical seeding, the probability that a null streamline encounters a nearby cortical segment by chance and is subsequently detected as a count, without having gone over the sulcal walls, is higher and thus, results in more shorter streamlines (Supplementary S1c). This means that the null tractography method likely overestimates null counts for connectivity mapping in the superficial white matter.

## Conclusion

We applied our recently developed *in vivo* method for mapping the SAF to map the SAF connectivity patterns in multiple cortical areas in the human early visual processing stream. The method was validated using the retinotopic organisation principle and robust retinotopic maps of the visual cortex obtained using fMRI *in vivo*. Our study demonstrated the applicability of our method beyond the connections of two cortical regions and represents a crucial step towards a comprehensive map of SAF connectivity patterns across the human brain.

## Supplementary Material

MovahedianAttar_et_al_Supplementary_materials_bhae279
