# Supplementary material for: Mapping short association fibre connectivity up to V3 in the human brain in vivo
Source: Cereb Cortex. 2024 Jul 24;34(7):bhae279. doi: 10.1093/cercor/bhae279 (PMC11267744; doi:10.1093/cercor/bhae279)
Supplement: MovahedianAttar_et_al_Supplementary_materials_bhae279 [file movahedianattar_et_al_supplementary_materials_bhae279.pdf]

S Supplementary Materials

S.1 Group streamline count matrices for null and ODF tractography

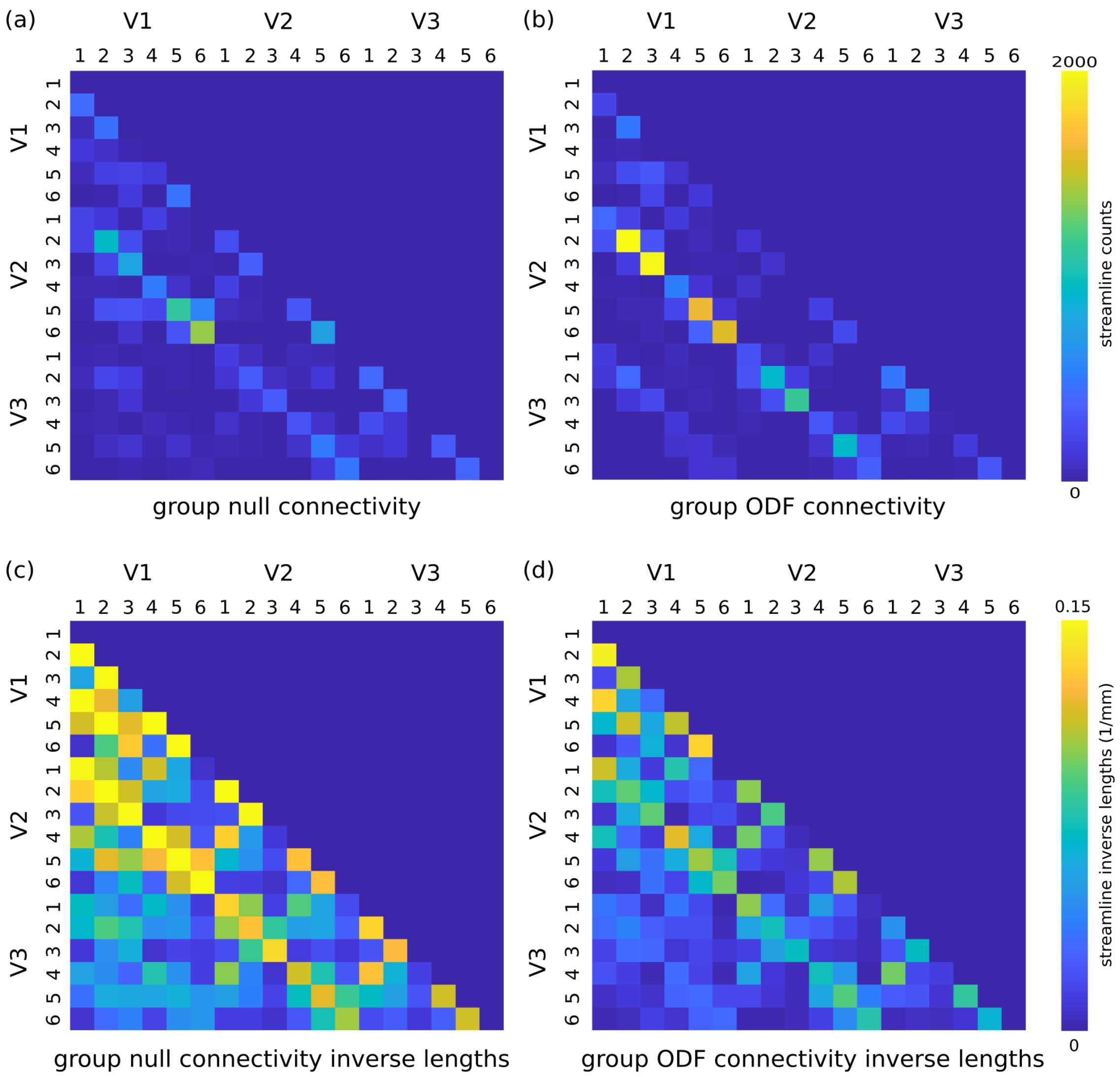

Fig. S1: Group-averaged matrices of streamline counts from null and ODF tractography and their corresponding inverse lengths. (a) Null distribution connectivity patterns exhibit some retinotopic order especially for the shorter streamlines connecting the nearest-neighbour V1–V2 and V2–V3 cortical areas (ratio of total retinotopic and non-retinotopic counts was 1.56 for V1–V2 and 1.48 for V2–V3). (b) Many more ODF streamlines were detected on-average than null streamlines, especially for the V1–V2 and V2–V3 connections (ratio of 3.57 for V1–V2 and 2.34 for V2–V3 ODF streamline counts). V1–V3 streamline counts were more comparable, yet the retinotopic patterns were more pronounced for ODF tractography, as expected (ratio of 0.70 for null and 1.45 for ODF streamline counts). The lengths of (c) null streamlines were on average shorter than the (d) ODF streamlines, pointing to the additional contribution of cortical geometry to the null streamline counts in nearby cortical areas.

S.2 Comparing our SAF mapping to results from a fibre architecture atlas

We qualitatively compared our SAF mapping using a high-resolution pipeline to a superficial white matter bundle atlas created based on DWI from the Human Connectome Project (HCP) dataset (Labra-Avila et al. 2020). The atlas mapped reproducible SAF as clusters of group probability maps created based on common anatomical locations of tractography-generated SAF streamlines across the brains of 76 participants. Direct quantitative comparison against our connectivity maps was not possible because our streamline clustering and averaging was done based on functional specialisation instead of anatomical organisation of SAF determined by functional retinotopic mapping. We performed a qualitative comparison by visualisation.

We identified SAF clusters containing V1, V2 and V3 connections in the atlas. Desikan cortical labels (Desikan et al. 2006) were used to designate the SAF. We used the V1, V2 and V3 cortical labels from the Jülich-Brain atlas (v3.0.3)(Amunts et al. 2023; Evans et al. 2012; Eickhoff et al. 2005) to identify the SAF connecting them. However, we reported these using their original Desikan labels because the spatial overlap between adjacent clusters made it challenging to specifically assign them to V1–V2, V2–V3 and V1–V3 connections (Fig. S2). For comparison, we created visualisations of SAF connecting distinct retinotopic sub-areas within V1–V2 for six representative hemispheres (three participants) in our dataset (Fig. S3).

We observed qualitative agreement between SAF mapped in the HCP atlas and our dataset. We could detect SAF clusters containing connections between V1, V2 and V3 in the atlas, which corresponded roughly to the anatomical locations and organisation of the SAF mapped in our dataset. A representative subset of the HCP SAF connecting areas within and between the lingual gyrus (Li), lateral occipital cortex (Lo) and cuneus (Cu) is shown in Fig. S2.

Similar to our results, both shorter (nearest-neighbour e.g., Fig. S2a,e,g,h) and longer (next-neighbour e.g. Fig. S2c,g and dorsal-ventral connection Fig. S2d) SAF were mapped in the atlas. Specifically, the cluster in Fig. S2b appears to correspond to V1–V2 SAF based on its anatomical location. Connections within the lateral occipital cortex (Fig. S2e) appear to correspond to SAF connecting patches 1–1 (i.e. centre of the visual field, see Fig. S3) and connections within the lingual cortex (Fig. S2h) appear to correspond to V1–V2 (blue cluster) and V2–V3 (green cluster) SAF. Further, the anatomical arrangement of the clusters in Fig. S2a,g suggested their retinotopic organisation in agreement with our results (Fig. S3). Some of the smaller clusters may correspond to intra-area connections.

The HCP bundle atlas holds potential for SAF mapping and analysis. It is the first atlas to report also the shortest SAF connecting adjacent cortical areas across the brain. Previous atlases reported the longer SAF ($> 30\,\mathrm{mm}$) (Román et al. 2022). However, validation e.g. against higher resolution datasets is required especially for the shorter and more curved SAF connecting smaller gyri/sulci. These are more difficult to track using DWI tractography at lower spatial resolutions because of the more pronounced partial volume effects. Additional SAF clustering based on e.g., Brodmann Area labels would facilitate comparison to other studies.

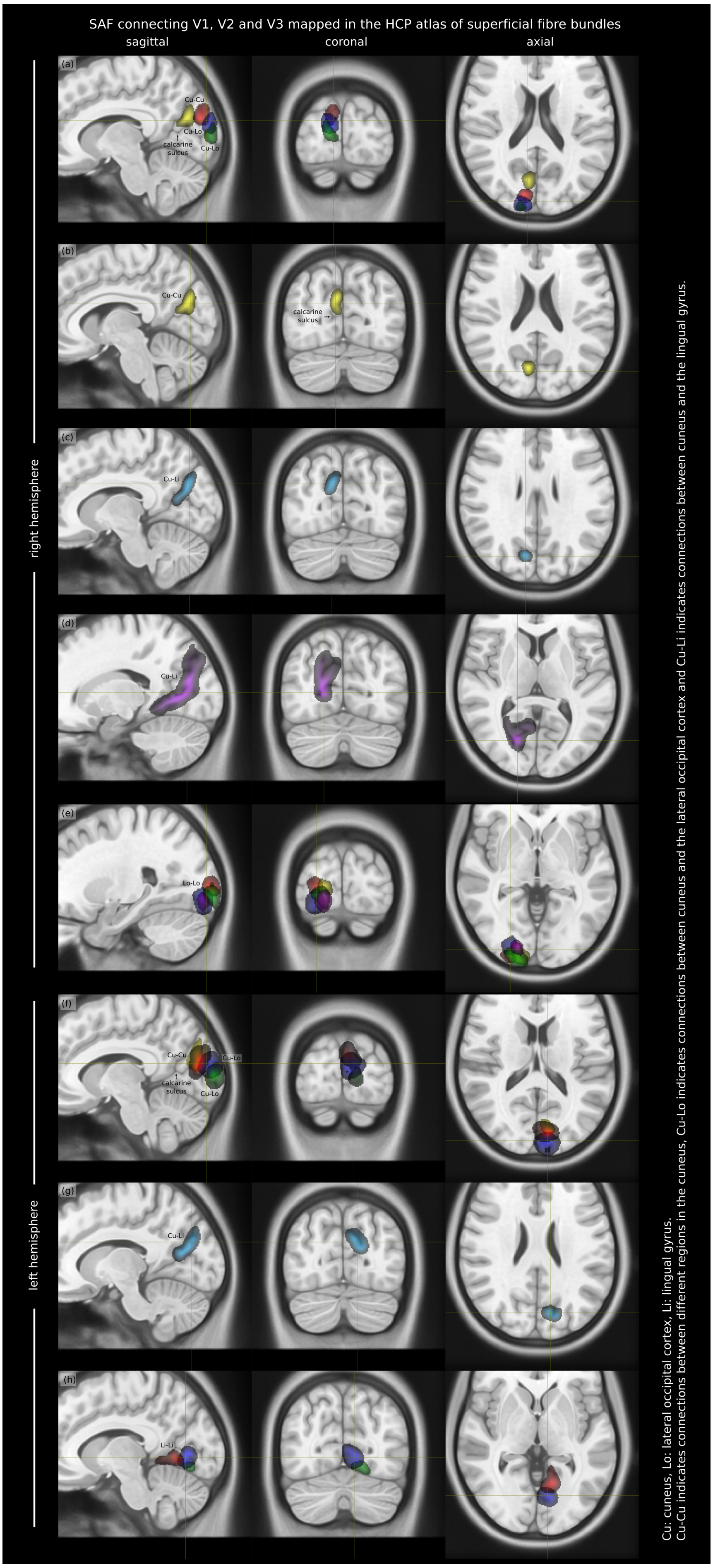

Fig. S2: A representative subset of HCP SAF bundles containing connections between V1, V2 and V3 shows qualitative agreement with SAF mapping in our dataset (see Fig. S3).

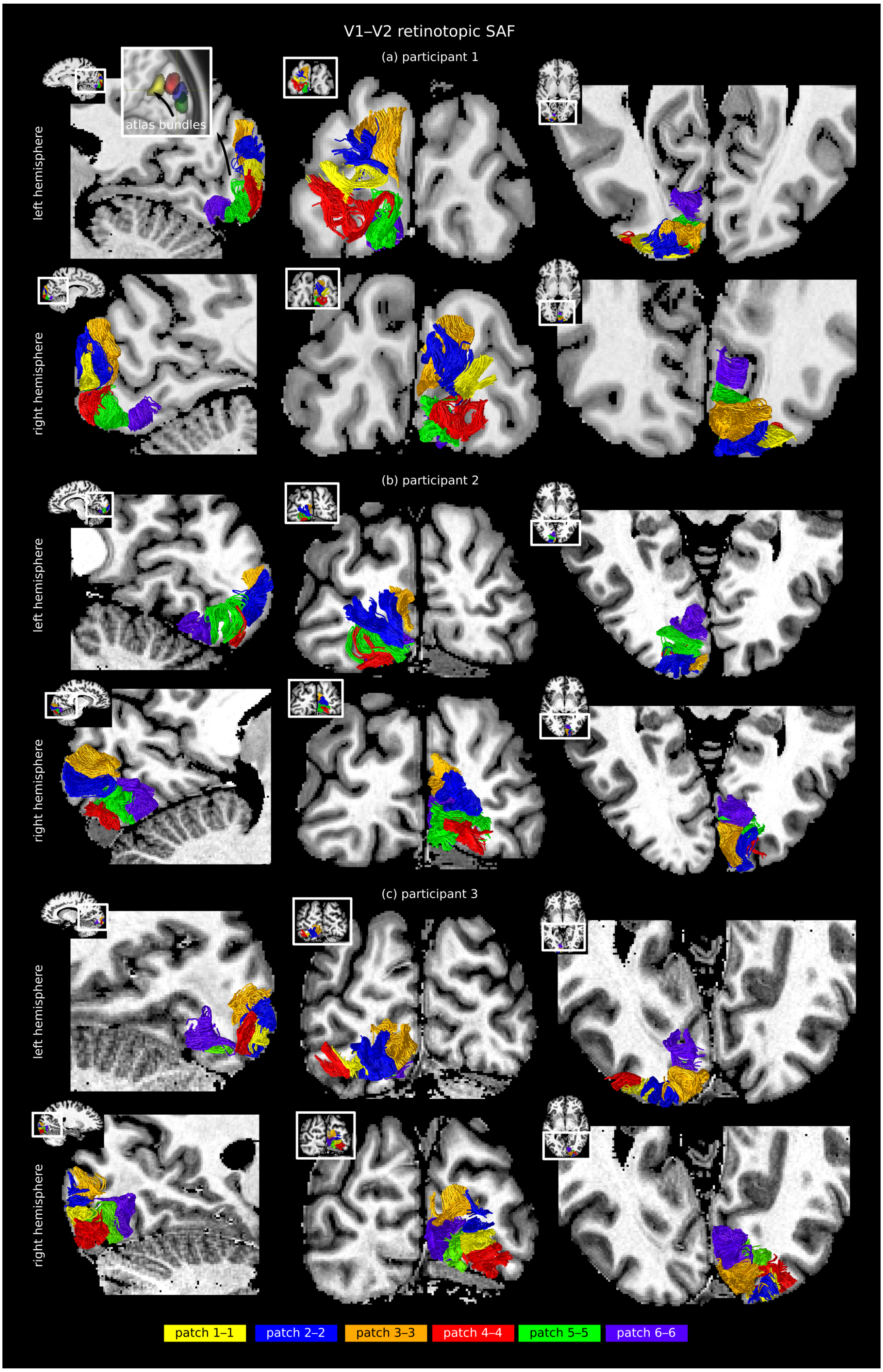

Fig. S3: V1–V2 SAF clustered into six functional sub-divisions based on functional maps of cortical retinotopic organisation for 3 representative participants. Topographic organisation of SAF clusters can be appreciated especially in sagittal view and shows qualitative agreement with SAF mapping in the HCP short bundle atlas (see Fig. S2a,f; inset from Fig. S2a and direction of arrows show topographic organisation and direction of increasing eccentricity, respectively).

S.3 Group-level estimates of cortical patch (sub-area) sizes

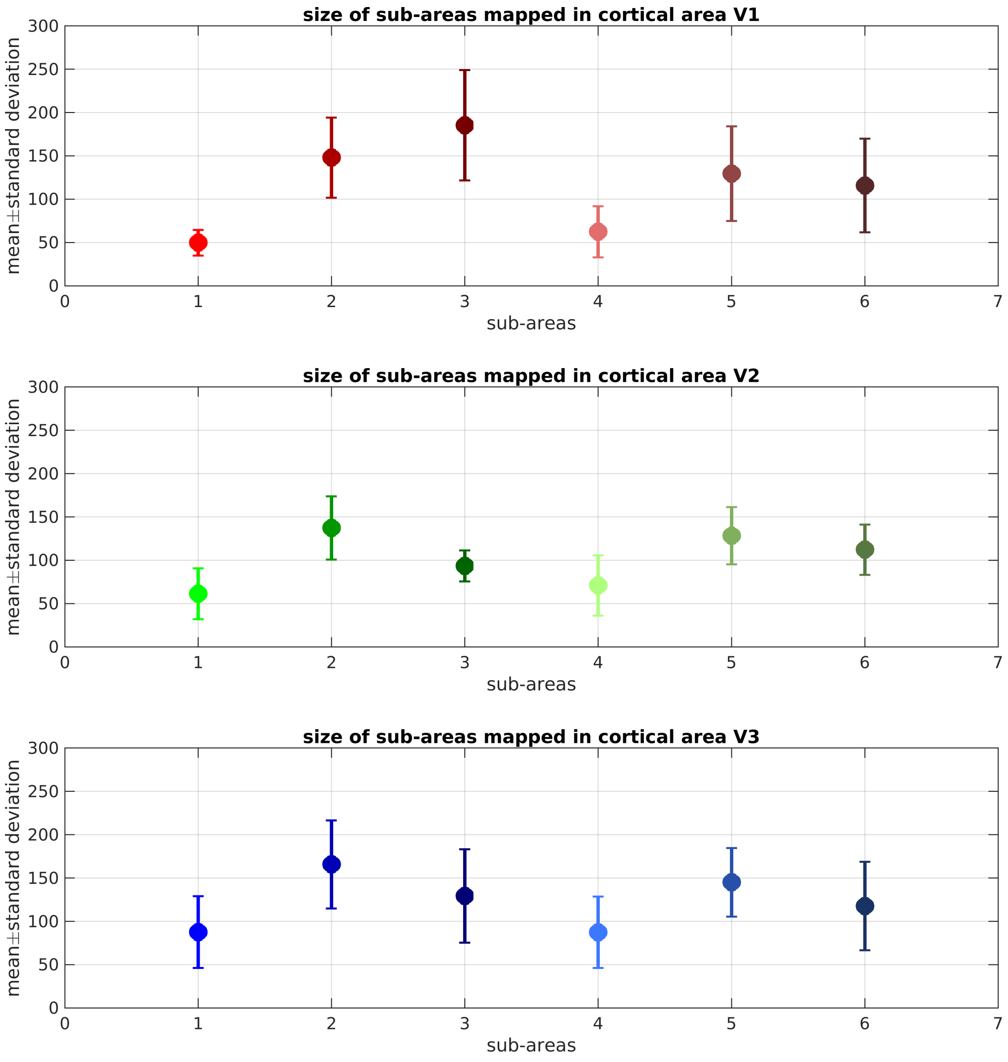

Fig. S4: Group-level estimates (mean±standard deviation) of the sizes (surface area in mm^2) of cortical sub-areas mapped by our fMRI retinotopic analysis setup in V1, V2 and V3 over all the hemispheres. The sub-areas were mapped using smoothed retinotopic maps. The sizes were computed as surface area covered on the cortex using FreeSurfer mris_anatomical_stats function. Group-average mean (circles) and standard deviation (error bars) were computed over all the hemispheres for V1, V2 and V3, separately. Sub-areas 1 and 4, corresponding to the cortical representations of the central (up to approximately 2° eccentricity in our experiments) visual field near the occipital pole (see also Fig. 1f) were relatively smaller. This is because the minimum eccentricity mapped in our fMRI experiment was 0.89°, meaning that sub-areas 1 and 4 occupied a 1° eccentricity range instead of the 2° covered by sub-areas 2, 3 and 5, 6. As a result, fewer streamlines were tracked by tractography, as also reflected in the lower connectivity indices in Fig. 3.
